# The Heart in Fabry Disease: Mechanisms Beyond Storage and Forthcoming Therapies

**DOI:** 10.31083/j.rcm2306196

**Published:** 2022-05-27

**Authors:** Maurizio Pieroni, Michele Ciabatti, Francesca Graziani, Antonia Camporeale, Elisa Saletti, Rosa Lillo, Stefano Figliozzi, Leonardo Bolognese

**Affiliations:** ^1^Cardiovascular Department, San Donato Hospital, 52100 Arezzo, Italy; ^2^Department of Cardiovascular Medicine, Fondazione Policlinico Universitario A. Gemelli IRCCS, 00168 Rome, Italy; ^3^Multimodality Cardiac Imaging Unit, IRCCS Policlinico San Donato, San Donato Milanese, 20097 Milan, Italy; ^4^Clinical Echocardiography Diagnostic Service, Cardio Center, Humanitas Research Hospital IRCCS, 20089 Rozzano, Italy

**Keywords:** Fabry disease, left ventricular hypertrophy, lysosomal storage, autophagy, unfolded protein response, myocardial inflammation

## Abstract

In patients with Fabry disease (FD), cardiovascular involvement is the main cause of death and reduction of quality of life. Left ventricular hypertrophy mimicking hypertrophic cardiomyopathy is the main feature of FD cardiac involvement although glycolipid storage occurs in all cardiac cellular types. Accumulation of lysosomal globotriasylceramide represents the main mechanism of cardiac damage in early stages, but secondary pathways of cellular and tissue damage, triggered by lysosomal storage, and including altered energy production, inflammation and cell death, contribute to cardiac damage and disease progression. These mechanisms appear prominent in more advanced stages, hampering and reducing the efficacy of FD-specific treatments. Therefore, additional cardiovascular therapies are important to manage cardiovascular symptoms and reduce cardiovascular events. Although new therapies targeting lysosomal storage are in development, a better definition and comprehension of the complex pathophysiology of cardiac damage in FD, may lead to identify new therapeutic targets beyond storage and new therapeutic strategies.

## 1. Introduction

Fabry disease (FD) is a rare, X-linked, inherited lysosomal storage disorder caused by pathogenic variants in the α-galactosidase A gene (*GLA*), resulting in complete or partial deficiency of the α-galactosidase A (α-Gal A) enzyme activity [1]. The enzymatic deficit leads to a progressive accumulation of lysosomal globotriasylceramide (Gb3) and related globotriaosylsphingosine (lyso-Gb3) in affected tissues, including heart, vessels, kidney, and peripheral nervous system [2, 3, 4].

Fabry disease is pan-ethnic and, although reported incidence figures range from 1 in 40,000 to 1 in 117,000, the true prevalence may be underestimated [2] and varies in different geographic regions, as highlighted by newborn screening programs in Italy and Taiwan reporting a prevalence of up to 1:8800 newborns [5, 6].

Currently, over 1000 *GLA* variants have been identified [1, 2], and characterized as pathogenic, variants of unclear significance (VUS) or benign without clinical relevance [1, 2]. Nonsense variants and stop-codons leading to absent or very low α-Gal A enzyme activity, are usually associated with the ‘classic’ early-onset FD phenotype, characterized by multiorgan involvement. Conversely, most missense variants allowing for residual α-Gal A activity, cause a late-onset phenotype, predominantly affecting the heart. In particular, the missense genetic variants p.N215S, p.F113L (prevalent in Portugal), and IVS4+919G>A (c.936+919G>A) (prevalent in Taiwan and southern China) are associated with prevalent cardiac disease [7, 8].

In the last 5 years a widespread application of high throughput next generation sequencing for the screening of high-risk patient cohorts, has led to identify many *GLA* VUS. To determine the pathogenic nature of these variants, clinical, biochemical, and histopathological evidence of FD is mandatory. Accordingly, some variants previously considered pathogenic have been recently reclassified as benign polymorphisms, as no evidence of Gb3 storage in target tissues could be demonstrated [9].

In female patients, random inactivation of the X chromosome in each cell during embryonic development (lyonization) results in a mosaic pattern with some cells expressing the normal allele and others the mutated one [10], leading to substantial variability in clinical phenotypes. The classic phenotype is associated with early symptoms starting in childhood and a rapid disease progression with clinical manifestations affecting the heart, kidney, and central nervous system [1, 2, 3, 4, 5]. In most heterozygous female patients, clinical manifestations range from an asymptomatic or mild phenotype affecting one or more organs and manifesting later in life, to a severe phenotype resembling that of males with classic FD [10].

Prenatal and neonatal histopathology studies demonstrated that Gb3 accumulations occur in fetal renal, myenteric plexus, and liver cells [11]. Similarly, myocardial damage starts early in life and progresses sub-clinically before significant signs and symptoms occur.

Overt cardiac involvement is represented by left ventricular hypertrophy (LVH) mimicking hypertrophic cardiomyopathy (HCM). Indeed, timely diagnosis is often missed, delayed or mistaken for other forms of hypertrophic cardiomyopathy (HCM) [3, 4]. Accordingly, a prevalence of *GLA* mutation of 0.93% in males and 0.90% in females has been recently reported among patients with a diagnosis of HCM [12].

Cardiac involvement represents the most common cause of death and reduction in quality of life in both male and female patients with FD [2, 3, 13], and represents an under-recognized cause of heart failure and ventricular arrhythmias in men aged >30 years and women aged >40 years [14]. However, the introduction of FD-specific therapies significantly modified the natural history of the disease with early diagnosis becoming essential to slow the progression or even prevent the development of cardiac and non-cardiac damage [2, 3, 15].

Recent progresses in understanding the mechanisms underlying progressive cardiac damage have reshaped the pathophysiology of cardiac involvement in FD, emphasizing the relevance of secondary storage-triggered cellular pathways. The therapeutic approach and the expected response to currently available therapies, have been also modified accordingly. The evolving treatment landscape aiming to prevent glycolipid storage would likely require, in a next future, the association of complementary treatments targeting additional intracellular and tissue-specific damage pathways.

This review article aims to provide an accurate and up-to-date review of current knowledge on the pathophysiology of cardiac damage and on the currently available and emerging therapeutic strategies for cardiac involvement in FD.

## 2. Pathophysiology of Fabry Disease Cardiac Manifestations

### 2.1 Classic Pathophysiology of Cardiac FD

**Fig. 1.**
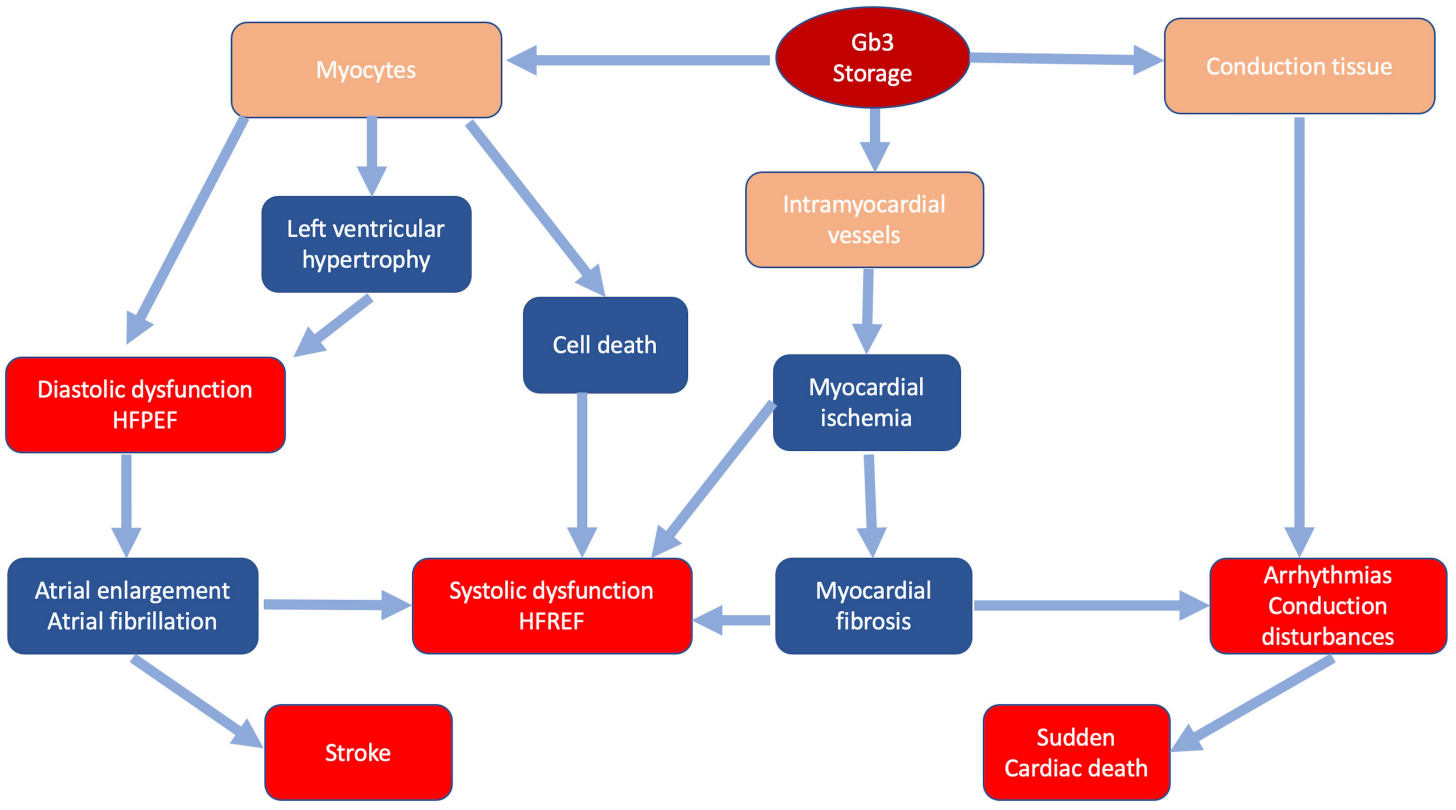
**Classic pathophysiology of cardiac involvement in Fabry disease**. In blue the main pathophysiologic mechanisms; in red the main clinical manifestations.

In FD storage of Gb3 occurs in all cardiac cellular types: myocytes, endothelial and smooth muscle cells of intramyocardial vessels, endocardium, valvular fibroblasts and conduction tissue [16, 17]. Intracellular glycosphingolipids organize in concentric lamellar bodies (zebra bodies) causing engulfment of the cytoplasm and cellular enlargement. Storage in myocytes causes a mechanical impairment, initially detectable only through advanced echocardiographic techniques as subclinical diastolic and systolic dysfunction [18]. Intracellular storage leads to cardiac wall thickening resulting in overt LVH that can mimic HCM. Progression of LVH and worsening of diastolic function can lead to further remodeling, including atrial enlargement and atrial fibrillation [19, 20]. Storage in intramyocardial vessel walls causes structural and functional changes leading to ischemia and ultimately replacement fibrosis with possible progression to systolic dysfunction [20]. Fibrosis and involvement of conduction tissue represent the substrate of ventricular arrhythmias and conduction disturbances [21] (Fig. 1). Valvular involvement, rarely severe, may contribute to disease progression and development of symptoms.

Right ventricular involvement is common in FD and mainly represented by right ventricular hypertrophy and speckle-tracking abnormalities, rarely associated with overt right ventricular dysfunction, at variance with other infiltrative disorders like cardiac amyloidosis with comparable degree of right ventricular wall thickening [22, 23].

### 2.2 Secondary Pathways of Cellular Damage beyond Storage

**Fig. 2.**
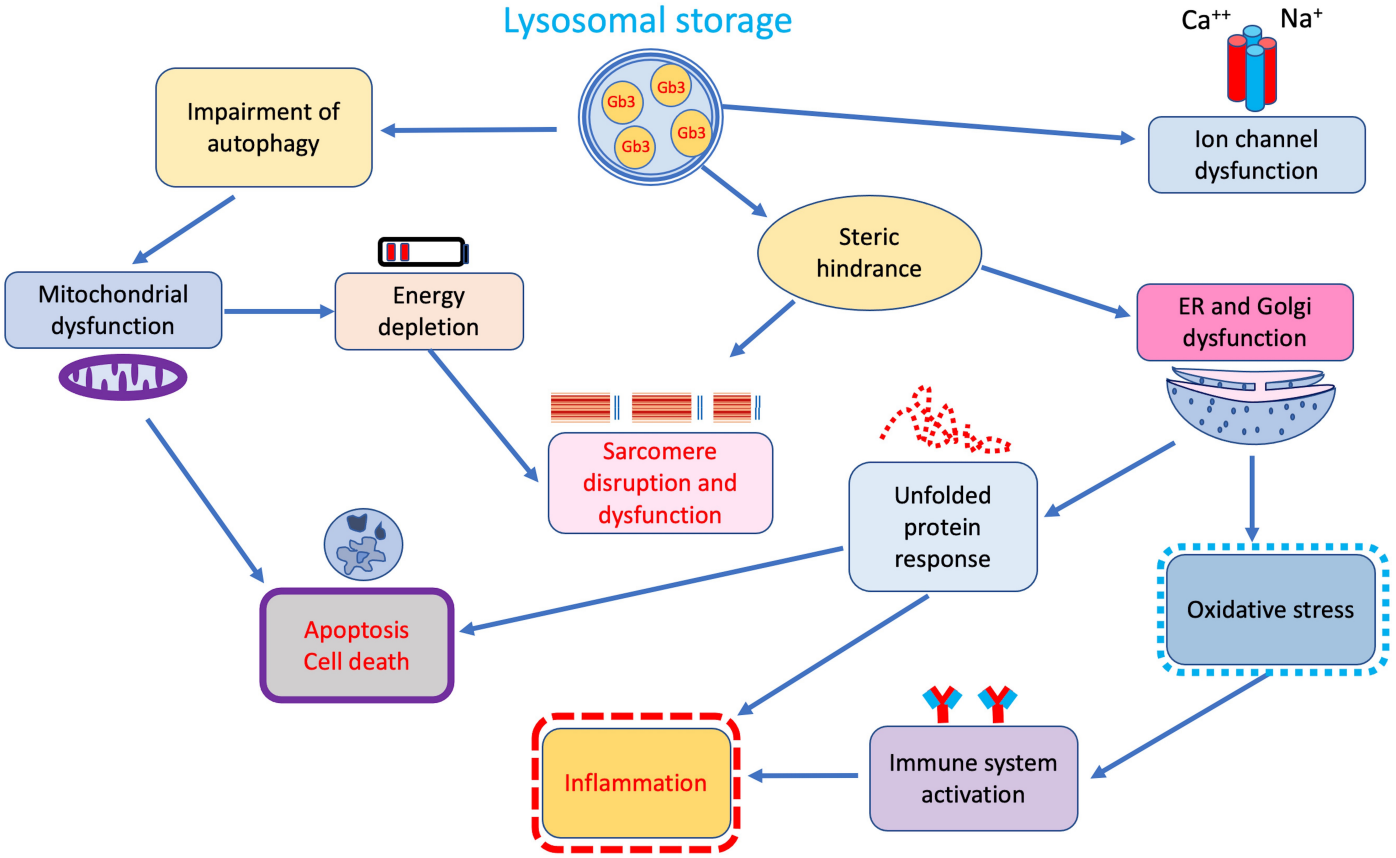
**Additional secondary cellular and tissue pathways of damage triggered by lysosomal storage**. ER, endoplasmic reticulum.

Although accumulation of Gb3 in lysosomes represents the main pathophysiological mechanism in FD, growing evidence indicates that secondary pathways of damage, activated by tissue-infiltrating and circulating glycosphingolipids, play a central role in the pathophysiology of the disease (Fig. 2) [24].

Indeed, a significant correlation between duration and intensity of lysoGb3 lifetime exposure and overall disease burden has been reported in both male and female patients with classic FD [25] suggesting that beyond storage, lysoGb3 may also represent a pathogenic factor [26, 27]. In fact, similarly to what has been observed in other inherited glycosphingolipidoses* in vitro* studies showed that intra-lysosomal Gb3 storage through steric hindrance may disrupt several lysosomal functions, including endocytosis and autophagy, thus interfering with mitochondrial energy production, and triggering apoptosis [28, 29, 30] (Fig. 2).

The autophagy–lysosome pathway (ALP) is an essential recycling pathway regulating cell survival and programmed death. Disruption of this pathway is a common feature of lysosomal storage disorders, including FD [31, 32]. Accordingly, pathology studies on cardiac biopsies from patients with different disease severity revealed increasing rates of cell death with subsequent development of replacement fibrosis [33].

In addition, glycolipid accumulation may induce an oxidative damage of DNA, myofibrils, and mitochondria leading to degradation of contractile elements, reduced ATP synthesis, and cell death [34, 35]. These mechanisms are probably responsible for the increased passive and decreased active forces described in isolated Fabry cardiomyocytes [35].

Steric hindrance is also responsible for the altered mitochondrial function and abnormal energy production processes reported in lysosomal storage disorders. Lücke and colleagues [36] observed a significant reduction of energy metabolism and mitochondrial functions, namely lower activities of respiratory chain enzymes I, IV, and V, in cultured fibroblasts from FD patients. Reductions in adenosine diphosphate ADP, adenosine monophosphate AMP and adenosine triphosphate ATP were also observed. Accordingly, 31(P)-CMR-spectroscopy demonstrated energy depletion of myocardial tissue in FD patients with LVH. Interestingly, in the same study energy metabolism improvement following enzyme-replacement therapy (ERT), preceded LVH regression [37]. Energy depletion coupled with the action of trophic factors like sphingosine, are thought to activate pathways of cellular hypertrophy common to sarcomeric HCM and other phenocopies [38].

Dysfunction of the endoplasmic reticulum represents another consequence of steric hindrance determined by Gb3 lysosomal storage. Consequences of endoplasmic reticulum dysfunction are mainly represented by release of oxidative stress products and mostly by induction of the unfolded protein response observed in cells of FD patients [39].

Unfolded protein response regulates many components of the secretory pathway to restore protein homeostasis, including protein folding, maintenance of calcium, but also inflammasome activation thus representing a well-established pro-inflammatory trigger [40]. Besides interference with intracellular organelles functions, Gb3 storage seems to also affect cell membrane structures. Birket and colleagues [41] demonstrated enhanced function of sodium and calcium channels, resulting in higher and shorter spontaneous action potentials, in FD cardiomyocytes obtained from induced pluripotent stem cells. These findings are in line with similar results on neuronal ion channels, thus suggesting that stored glycosphingolipids may alter ion channel expression and/or cell membrane trafficking, interfering with the electrical properties of cardiomyocytes [42]. Indeed, Namdar and colleagues [43] hypothesized that an increased conduction velocity in atrial and ventricular myocardium may underlie the electrocardiographic abnormalities in FD, including the presence of a short PR interval in the absence of an accessory pathway.

Finally, when considering the complexity of myocardial pathophysiology in FD, it should be emphasized that patients with the same gene variant, even in the same family, may present different severity of clinical manifestations, suggesting that also other genetic factors, like the presence of additional *GLA* variants, concomitant variants in other genes and environmental factors, may influence the phenotypic expression [44]. With this regard, it’s worth to mention that in patients with an established diagnosis of FD, in the presence of significant LVH in children and adolescents, severe LVH in young adults or evidence of male-to-male transmission of LVH phenotype, the presence of concomitant sarcomeric gene mutations should be ruled out, particularly when response to FD-therapy is scarce or null [45]. Similar secondary mechanisms of damage mediated by Gb3 and lysoGb3 beyond the lysosomal storage, have been demonstrated also in other affected tissues. In arterial vessels, lysoGb3 promotes smooth muscle cell proliferation, causing wall remodeling with increase of intima media thickness and arterial stiffness of small and large arteries, including myocardial and renal vessels [46]. In addition, lysoGb3 at concentrations consistent with levels detectable in FD patients, can impair endothelial nitric oxide synthase, further contributing to the widespread vasculopathy observed in Fabry patients, particularly in classic FD [47, 48].

In kidneys, lysoGb3 is thought to contribute to podocyte loss and glomerulus fibrosis through different mechanisms, triggering apoptosis and inflammatory activation similarly to what described for myocardial tissue [49, 50]. With this regard it should be reminded that both cardiomyocytes and podocytes are terminally differentiated cells with very low, if any, rates of turn-over. A recent proteome analysis study showed that α-Gal A-deficient podocytes present dysregulation of proteins involved in lysosomal trafficking and function, metabolism and thermogenesis regulation, cell-cell interactions and cell cycle. Of note treatment with induction of α-Gal A expression normalized protein expression only in part. A similar abnormal protein expression was also observed in endothelial and epithelial cells, suggesting a FD-specific rather than a cell-specific intracellular pathways disruption [51].

At neuronal level it has been demonstrated that lysoGb3 at concentrations detectable in plasma of FD males, damages nociceptive neurons, likely accounting for the reported small-fiber neuropathic pain affecting classic FD patients [52]. Similarly, duration of exposure to lysoGb3 significantly correlated with thermal sensory limen and the cold detection threshold of hand and arms [53].

### 2.3 The Role of Inflammation

Increasing evidence supports a role of inflammation in early pathogenesis and progression of cardiac damage [50, 54]. Accumulation of Gb3 and lyso-Gb3 can trigger a chronic inflammatory response either promoting the release of—or behaving themselves as—damage mediators [55] (Fig. 1). Altered peptides derived from oxidative stress or from abnormal enzymes digestion may act as neoantigens activating the immune system. Unfolded protein response, as previously mentioned is also a potent trigger of inflammatory response. Glycolipids can act themselves as antigens when presented to natural killer T cells. The Gb3-mediated effects can be abolished by antibodies blocking the toll-like receptor 4, demonstrating a pivotal role of this inflammatory pathway [50, 54]. Activation of toll-like receptor 4 pathway may also enhance TGF-β response leading to remodeling of the extracellular matrix and myocardial fibrosis [49].

Endomyocardial biopsy studies showed the presence of myocarditis in up to 56% of patients with FD cardiomyopathy [56]. In the presence of positive antiheart and antimyosin antibodies and negative polymerase chain reaction for viral genomes, an immune mediated process was supposed and a significant correlation with disease severity was observed suggesting that myocardial inflammation may contribute to progression of FD cardiac damage and resistance to ERT.

In addition, signs of systemic and myocardial chronic inflammatory activation are consistently detected in patients with FD [57].

The widespread use of new cardiac magnetic resonance (CMR) techniques, namely T1 and T2 mapping, further supported the view of FD as a storage/inflammatory disease providing a qualitative assessment of myocardial tissue in terms of myocardial lipid content and inflammation.

Systematic evaluation of patients with different gender, age and type of disease (classic or late-onset) led to identify sequential phases of cardiac damage evolution [58, 59, 60] suggesting a three-stage progression of Fabry cardiomyopathy: an initial phase, starting since childhood, characterized by myocardial storage without signs of inflammation nor overt LVH. In the second phase, secondary pathways particularly inflammation and hypertrophy take place leading to clinical manifestations and ECG and imaging abnormalities. In this phase T1 lowering, indicative of myocardial glycolipid storage and T2 elevation indicative of myocardial edema/inflammation, may precede LVH, mostly in female patients. Markers of this phase, characterized by myocardial inflammation and injury, are the release of troponin and an initial increase of NT-proBNP. In the absence of specific therapy, cardiac damage progresses towards severe LVH and fibrosis, precipitated by concomitant mechanisms, in particular myocardial ischemia. Higher NT-proBNP levels are typical of this phase, characterized by overt symptoms and poor response to FD-specific therapies. Myocardial perfusion impairment is another early feature of cardiac involvement, likely reflecting microcirculation dysfunction induced by storage and inflammation. Whether myocardial inflammation is the main mechanism leading to myocardial fibrosis remains to be fully elucidated. It remains also unclear whether secondary pathways triggered by Gb3 storage, including systemic and tissue inflammation, may become storage-independent and thus resistant to FD-specific therapies aimed at halting or preventing further Gb3 storage.

## 3. Management of Cardiac Fabry Disease

The increasing recognition of additional pathophysiologic pathways operating in the pathogenesis of cardiac damage in FD, while allowing a better comprehension of the potential pitfalls of currently available treatments, also paves the way for the development of additional therapeutic strategies with targets beyond Gb3 storage prevention.

The main therapeutic goal of FD treatment is to halt or at least to slow the progression towards irreversible tissue damage and organ failure, thus preventing major cardiovascular events [61]. According to a recent European expert consensus statement on therapeutic goals in Fabry disease [62], the impact of any FD-specific therapy depends upon patient- and disease-specific factors and timing of initiation, considering that the disease spectrum ranges from classic early-onset disease to non-classic later-onset phenotypes, and that complications occur in multiple organs or are confined to a single organ depending on the stage of the disease. In this document, a series of organ-specific treatment goals and patient management algorithms are proposed, considering inter-patient differences in disease severity, natural history, and treatment responses as well as the negative burden of therapy and the importance of multidisciplinary care.

The statement, while emphasizing the need for early disease-specific therapy to delay or slow the progression of disease, also highlight the need for non-specific adjunctive therapies to prevent or treat the effects of organ damage on quality of life and long-term prognosis.

Then, a comprehensive management of cardiac FD should include FD-specific and cardiologic adjunctive therapies to prevent major cardiovascular events and manage cardiovascular symptoms.

## 4. FD-Specific Treatments

### 4.1 Currently Approved Therapies

Currently available FD-specific treatments include ERT (agalsidase alfa and agalsidase beta) and an oral pharmacological chaperone (migalastat). Both therapies are indicated in patients with an established diagnosis of FD, with the pharmacological chaperone indicated only in those patients with an amenable variant.

Long-term follow-up studies and registry data demonstrated that ERT may halt or slow progression of cardiac disease reducing the rate of cardiovascular events, particularly when started early [2, 15]. Regression of mild LVH has been reported in patients with the classic and cardiac phenotype, with some evidence that LVH may be prevented by early treatment [61]. In a recent study ERT-naïve patients presented attenuated T1 lowering, with small reductions in maximum wall thickness and stabilized LVMI after 1 year of ERT [63].

When cardiac involvement is advanced, response to ERT is limited [61, 63] and there is evidence that despite 1-year-ERT an increase of T2 signal and LGE area with worsening global longitudinal strain can be observed at CMR.

Endomyocardial biopsy studies have shown that ERT reduces endothelial Gb3 inclusions in myocardial vessels, while clearance of Gb3 from cardiomyocytes is less significant [64]. The development of neutralizing antibodies binding exogenous enzyme molecules and preventing them to reach target cells, is among the possible causes for a limited efficacy of ERT in some patients [65].

Chaperone molecules are iminosugars that in amenable variants of FD bind to the catalytic domain of α-Gal A promoting its proper folding and trafficking to the lysosome, thus increasing enzymatic activity and Gb3 degradation. The same molecules at higher doses exert an inhibitory action on α-Gal A. Amenable *GLA* variants are defined by an increase by at least 20% of enzymatic activity in patients’ lymphocytes cultured with 20 mM Migalastat.

A list of *GLA* variants resulted amenable *in vitro* is available online in a public online database. Clinical trials and open-label extension studies have shown that treatment with Migalastat is associated with a small but significant decrease in indexed left ventricular mass assessed by echocardiography [66]. However, no CMR data on Migalastat effects on myocardial damage are currently available. In addition, recent real-world studies demonstrated that in some genetic variants a significant discrepancy between predicted *in vitro* amenability and the effective *in vivo* increase in α-Gal A activity and clinical response can be observed [67]. These pitfalls of Migalastat treatment may recognize several causes including the possible dosage-dependent inhibitory effects of Migalastat and the intrinsic limitations of the *in vitro* amenability test. Accordingly, a careful monitoring of the biochemical and clinical response to chaperone therapy is required when treatment is initiated to confirm clinical efficacy.

### 4.2 Therapies under Development

New therapies under development are represented by second-generation ERTs, substrate reduction therapies and gene and mRNA therapies [5, 68].

New generation ERTs include plant-derived ERTs developed to reduce ADAs formation and improve enzyme biodistribution. Pegunigalsidase alpha is a novel pegylated form of α-Gal A produced in a PlantCell Ex system (Protalix Biotherapeutics, Carmiel, Israel) characterized by a much longer circulatory half-life and increased heart and kidney uptake compared to currently available ERTs.

In the phase III BRIDGE trial (NCT03018730) patients switched from agalsidase alfa to Pegunigalsidase alfa showed stabilization, or at least slower progression of kidney failure (eGFR slope improved from –5.1 to 0.23 mL/min/1.73 $m^{2}$/year in both male and female) [69]. Other clinical trials (NCT02795676; NCT03018730, NCT03180840) evaluating Pegunigalsidase alfa treatment are still ongoing,

Substrate reduction therapy (SRT) is represented by oral iminosugars blocking the glucosylceramide synthase enzyme thus inhibiting glycosphingolipid synthesis to lower the cellular amount of Gb3. This therapeutic approach, already validated in Gaucher disease, can be administered irrespective of genotype. There are two SRT formulations, venglustat and lucerastat, currently under investigation in phase II and III clinical trials respectively [70].

Both novel SRT agents are promising potential oral therapeutics for the nearby future with no limitation regarding specific mutations as seen in chaperone therapy.

In phase I/II clinical trials treatment with lucerastat led to a significant reduction of glycosphingolipids, glucosylceramide, lactosylceramide, and globotriaosylceramide plasma levels compared to baseline, with a good safety and tolerance profile [71]. A randomized multi-center double-blind clinical phase III study with lucerastat is currently ongoing.

Genetic therapy is considered the definitive treatment for many genetic disorders including lysosomal storage diseases. In the last decades, both *in vivo* and *ex vivo* gene therapy approaches have been explored. In a recent phase II clinical trial, hematopoietic stem cells were retrieved from patients, transfected with lentiviruses (AVR-RD-01, Avrobio) and re-administered to the patient showing a persistent elevation in α-Gal A activity [72]. However, despite encouraging safety and efficacy results this research line has been recently deprioritized by the pharmaceutical company.

*In vivo* approaches with liver targeted adenoviral-mediated gene transfer showed in preclinical studies with α-Gal A knockout mouse model, a significant increase of α-Gal A activity associated with a marked reduction of lyso-Gb3 [73]. Regarding this approach, it remains unclear whether the uptake of enzyme released by transfected cells by affected tissues will be sufficient to compensate the enzymatic deficit. Indeed, when considering the case of heterozygous females, cross-correction doesn’t seem able to restore adequate enzymatic activity and prevent Gb3 storage. In addition, it is also possible that male patients with classic FD and absent a-Gal A activity could develop ADAs against the expressed enzyme, as observed for ERT, although given the continuous exposure and endogenous synthesis and glycosylation, tolerance towards the new antigen could be expected in most of cases. To specifically target myocardial tissue, novel cardiac-tropic vectors have been developed and are currently tested in non-human primates. Initial studies suggest an increased gene delivery and a reduced immunogenicity compared with conventional adenoviral vectors. Very recent data in adult male patients with classic FD showed safety and efficacy of the vectors with evidence of biochemical efficacy (sustained increase of α-Gal A activity and significant decrease of lyso-Gb3 levels), together with promising cardiac improvement in terms of T1 mapping increase at CMR.

The administration of human α-GAL A mRNA encapsulated with lipid nanoparticles, has been also tested in mice and non-human primates showing a significant increase of α-GAL A levels in liver, heart, and kidney [74].

Cell transplantation represents another potential strategy to treat lysosomal diseases. In a recent study a new ex vivo gene therapy platform was developed using a transplant pack, consisting of a porous membrane and spheroids with scaffolds. These membranes have countless pores of less than 0.1 ${\mathrm{mm}}^{2}$ allowing secretion of proteins keeping them separated from the host immune system. The packs with cells overexpressing α-Gal A were subcutaneously transplanted into the backs of mice, leading to high levels of enzyme in plasma and livers. Compared to classic ERT this technique has several advantages including continuous enzyme secretion and potentially a one-time treatment to cure diseases [75].

In general, while initial experience and short-term results, are promising, the long-term efficacy and potential adverse effects of gene therapy and cellular therapies remain unclear and require larger studies with longer follow-up.

### 4.3 Conventional Cardiovascular Therapies

Cardiovascular complications are the first cause of morbidity and mortality in patients with FD. Therefore, conventional cardiovascular therapies including pharmacological and interventional strategies are essential to improve survival and quality of life in these patients. In a recent consensus document, expert recommendations have been provided regarding treatment and follow-up [76]. It is important to emphasize that FD does not represent a contraindication to conventional invasive therapies including percutaneous and surgical myocardial revascularization procedures or pacemaker and defibrillator implant. Similarly, interventional structural cardiology and radiofrequency ablation can be considered in FD patients with valvular disease or life-threatening arrhythmias, while patients with advanced heart failure can be proposed for cardiac transplant.

With this regard, sudden cardiac death prevention remains challenging in FD, particularly in patients with advanced cardiac involvement. Current recommendations for HCM, including the risk calculator developed by European Society of Cardiology, cannot be applied. Therefore, prognostic stratification relies on the identification of risk factors including advanced LV hypertrophy, extensive fibrosis, and unexplained syncope [77].

Similarly, considering the increased risk of stroke and also of cerebral microbleeds associated with FD, when considering stroke prevention strategies in patients with atrial fibrillation, classical scoring systems (i.e., CHA2DS2VASC and HAS-BLED) should not be applied. As patients with HCM, all patients with FD and atrial fibrillation should be considered candidates to oral anticoagulation. Although there are no studies assessing safety and efficacy of direct oral anticoagulants in FD, these drugs should be preferred (when not contraindicated for severe impairment of renal function) considering the augmented risk of intracranial hemorrhage and nephropathy associated with vitamin-K antagonists administration. It is also important to remind that amiodarone is a cationic amphiphilic molecule that may further worsen lysosomal pH and functions, potentially reducing the effect of ERT. Therefore, long-term treatment with this drug, either for ventricular arrhythmias or rhythm control in atrial fibrillation should be avoided or limited to selected cases with close monitoring of ERT effects [76].

## 5. Future Perspectives

In the last decades FD-specific therapies have significantly changed the natural history in terms of long-term survival and quality of life. On the other hand, the effects of these therapies on cardiac involvement appear still incomplete, as a significant impact on cardiac FD can be obtained only with early treatment, while clinical effects are more limited in advanced cases. Many factors may limit the efficacy of ERT on cardiac damage (anti-drug antibodies formation, lower concentrations and higher instability of administered enzyme in myocardial tissue, inability to clear terminally differentiated cardiomyocytes) while data on long-term cardiac efficacy of Migalastat are still lacking.

**Fig. 3.**
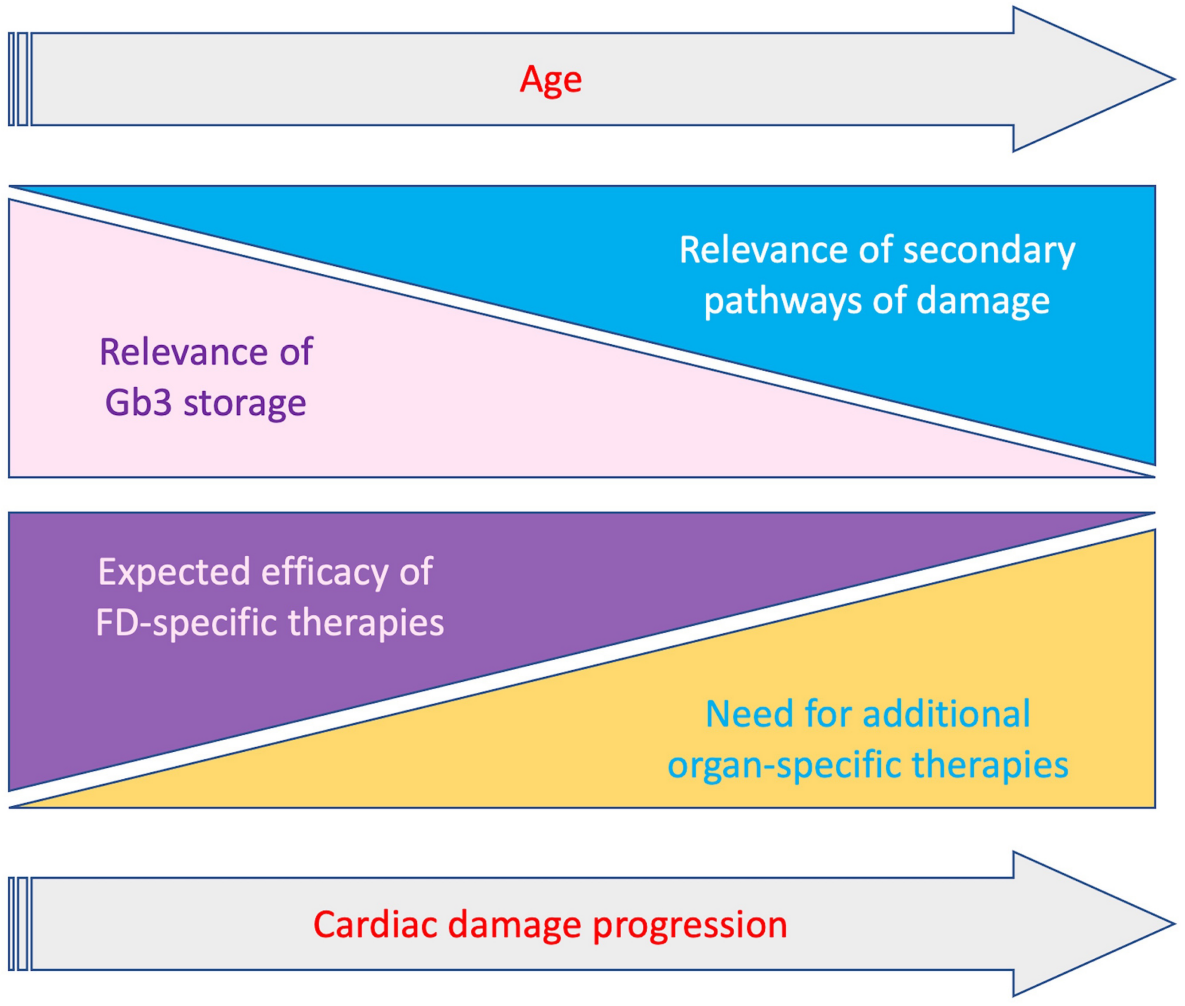
**Schematic representation of relevance of Gb3 storage and secondary mechanisms and clinical impact of FD-specific and organ-specific treatments, according to progression of cardiac involvement and age**. With the progression of cardiac damage the relevance of Gb3 storage decreases while secondary pathaways become prominent. Accordingly the impact of therapies targeting Gb3 storage gradually decrease with the need of additional organ specific treatments targeting secondary pathways.

Several strategies to optimize currently available therapies have been proposed, like immunosuppression to minimize the detrimental effect of anti-drug antibodies, or the co-administration of ERT and chaperones to improve stability and bioavailability of exogenous enzyme [78]. Nevertheless, the activation of the secondary pathways of damage previously described represents an additional component of cardiac damage progression despite FD-specific therapies, particularly in patients with overt cardiac involvement. Indeed, restoring lysosomal function could represent only a part of the treatment of this cardiomyopathy (Fig. 3). A better comprehension of the pathways triggered by lysosomal dysfunction and the possible development of specific therapies, appear essential to further improve the management of these patients. In particular, it will be important to clarify which pathogenic pathways may become storage-independent, thus representing alternative therapeutic targets. In this regard, the role of inflammation in both early and late stages of cardiac damage must be further investigated, also in therapeutic terms. In recent studies, pentosan polysulfate, a mixture of semisynthetic sulfated polyanions, showed anti-inflammatory activity in mucopolysaccharidosis type 2 patients, and reduced pro-inflammatory cytokine secretion in cultured peripheral blood mononuclear cells from patients with Fabry and Gaucher disease [79]. In this sense the use of cardiomyocytes derived from isolated pluripotent stem cells may also offer the opportunity to study genomic and proteomic changes occurring in early stages of the disease.

A deeper understanding of mechanisms of cardiac damage in FD may also provide insights for other cardiomyopathies and other non-cardiac conditions. Understanding the central role of defective lysosomal/endosomal transport has recently revealed links between Gaucher and Parkinson’s disease [80]. Additionally, the lysosomal protein NPC1, defects in which result in Niemann Pick disease, is also involved in the Ebola virus infection-replication cycle. On the other hand, new therapies targeting the pathophysiological mechanisms of HCM like myosin modulator mavacamten have been recently approved, opening the way to drugs interfering with the intracellular molecular pathways and potentially representing an additional therapeutic option also for FD-related cardiomyopathy [81].

## 6. Conclusions

Lysosomal storage of glycosphingolipids represents the main mechanism of cardiac damage in early stages of FD, while secondary pathways of cellular and tissue damage, triggered by lysosomal storage, contribute to cardiac damage and disease progression. The role of these mechanisms appear prominent in more advanced stages, hampering and reducing the efficacy of FD-specific treatments. Conventional cardiovascular treatments in addition to FD-specific therapies are necessary to manage cardiovascular symptoms and reduce cardiovascular events. Although new therapies aimed to halt or slow lysosomal storage or to correct the genetic defect are in development, a better definition and comprehension of the complex pathophysiology of cardiac damage in FD, is essential to identify new therapeutic targets beyond storage and new therapeutic strategies.
